# Papillary Carcinoma Within Thyroglossal Duct Cyst: A Rare Midline Coexistence

**DOI:** 10.7759/cureus.31906

**Published:** 2022-11-26

**Authors:** André De Sousa Machado, David Dias, Ana Silva, Luis Meireles

**Affiliations:** 1 Otolaryngology, Centro Hospitalar Universitário do Porto, Porto, PRT

**Keywords:** otolaryngology case report, sistrunk’s procedure, papillary carcinoma of thyroid, thyroglossal duct, thyroid cancer surgery

## Abstract

Thyroglossal duct cysts (TDCs) develop papillary carcinomas in very rare cases. Mostly, in such cases, the diagnosis is established after the excision of a clinically benign TDC. An anterior neck mass was found in a 43-year-old man with papillary carcinoma arising in a TDC. Clinical, radiological, and analytical controls are necessary for the management of papillary carcinoma in the TDC. There is no need for thyroidectomy unless there are palpable abnormalities in the gland or significant changes on an ultrasound. The outcome of the papillary carcinoma does not seem to be significantly impacted by routinely performed thyroidectomy.

## Introduction

Around 7% of adults are affected by thyroglossal duct cysts (TDCs), a congenital anomaly [1]. The tumor appears to originate in the foramen caecum from epithelial remnants of the thyroglossal tract. The most common location for TDCs is between the thyroid gland and the hyoid bone (61%), followed by the suprahyoid (24%), suprasternal (13%), and intralingual (2%) regions [2]. Usually, it displays as a bulge in the midline of the anterior cervical region, mobile with deglutition, and protrusion of the tongue. Only some cases of papillary carcinoma appearing in TDCs have been described in the literature. [1,2] We report such a clinical case with our respective approach.

## Case presentation

A 43-year-old male patient, with no relevant personal or family history, presented to our department with complaints of odynophagia within two months of evolution, associated with the appearance and progressive growth of cervical swelling in the midline. The patient denied any previous similar episodes and presented no other complaints. On physical examination, the patient presented an anterior cervical swelling, approximately 3.5 cm of the greatest axis, with hard-elastic consistency and mobile, with protrusion of the tongue, suggestive of a TDC. No other signs were found on ENT examination, including neck palpation and laryngoscopy.

**Figure 1:**
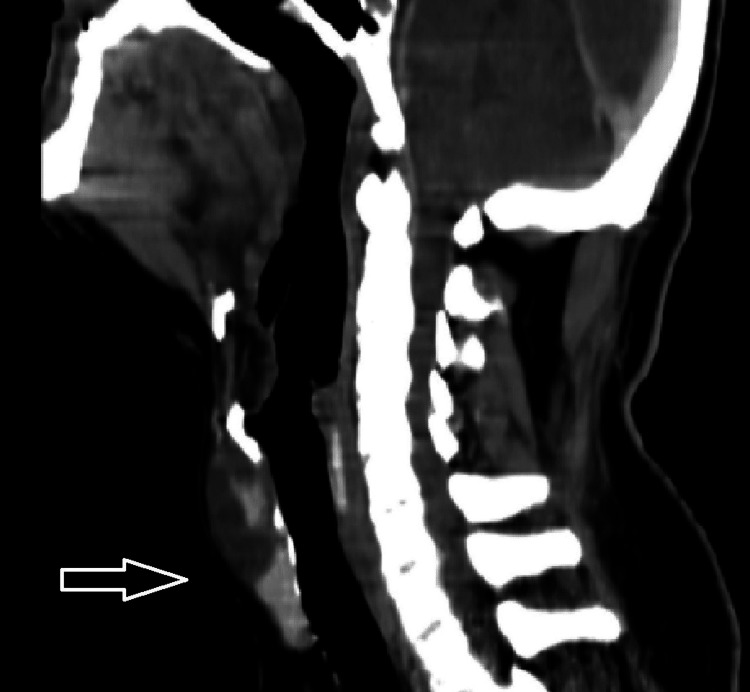
Preoperative CT scan image showing a well-defined midline infra-hyoid multicystic lesion, with slight capsular enhancement, compatible with a thyroglossal duct cyst (white arrow).

Ultrasound and cytopathological examination (the latter performed through fine needle aspiration (FNA)) were highly suggestive of TDC diagnosis, with no evidence of malignant cells. Ultrasound confirmed the presence of normal thyroid gland tissue with no clinically detectable disease, and no cervical adenopathies were found. A pre-surgical CT scan was also performed, which demonstrated a well-defined midline infra-hyoid multicystic lesion, anterior to the pre-epiglottic space of the larynx, with slight capsular enhancement, in favor of TDC diagnosis (Figure 1).

**Figure 2:**
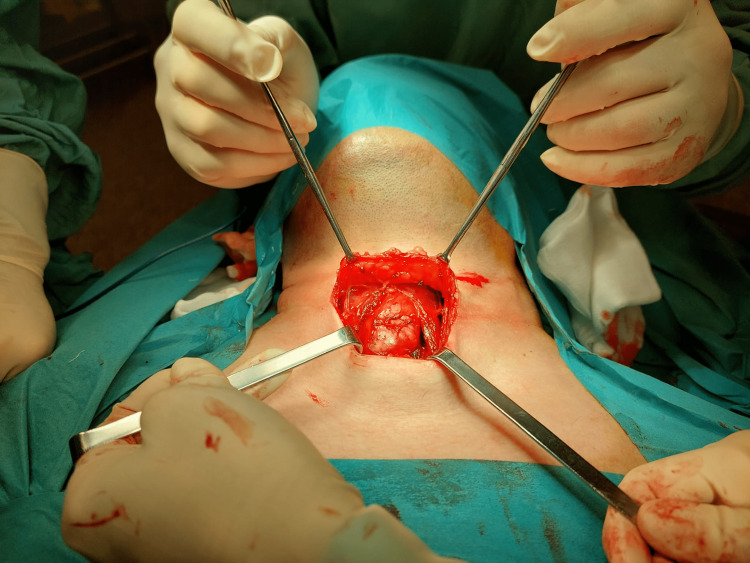
Sistrunk procedure. Intra-operative picture.

Surgical removal of the lesion was performed according to the principles of the Sistrunk procedure: excision of the cyst, the middle portion of the body of the hyoid bone, and a core of tissue around the thyroglossal tract until the foramen caecum (Figure 2). The surgery was uneventful.

**Figure 3:**
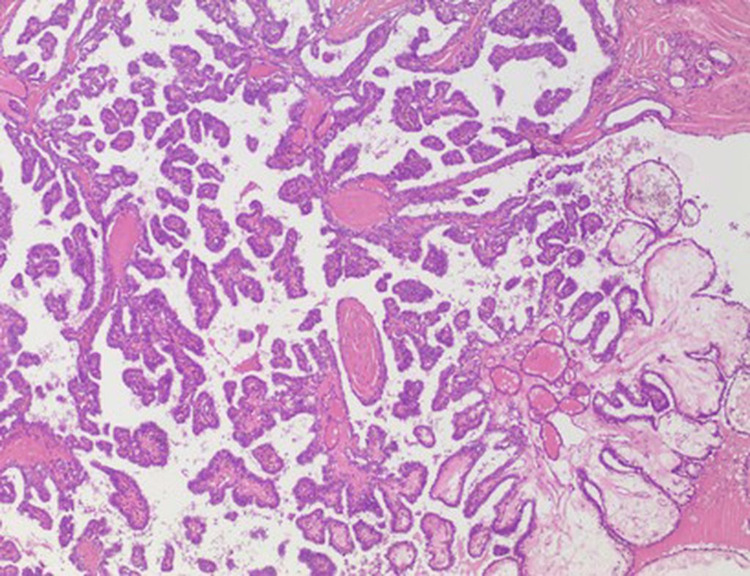
Papillary carcinoma, classic type with typical papillary pattern of growth and nuclear features of papillary thyroid carcinoma. No colloid of Psammoma bodies was present. 40x magnification.

**Figure 4:**
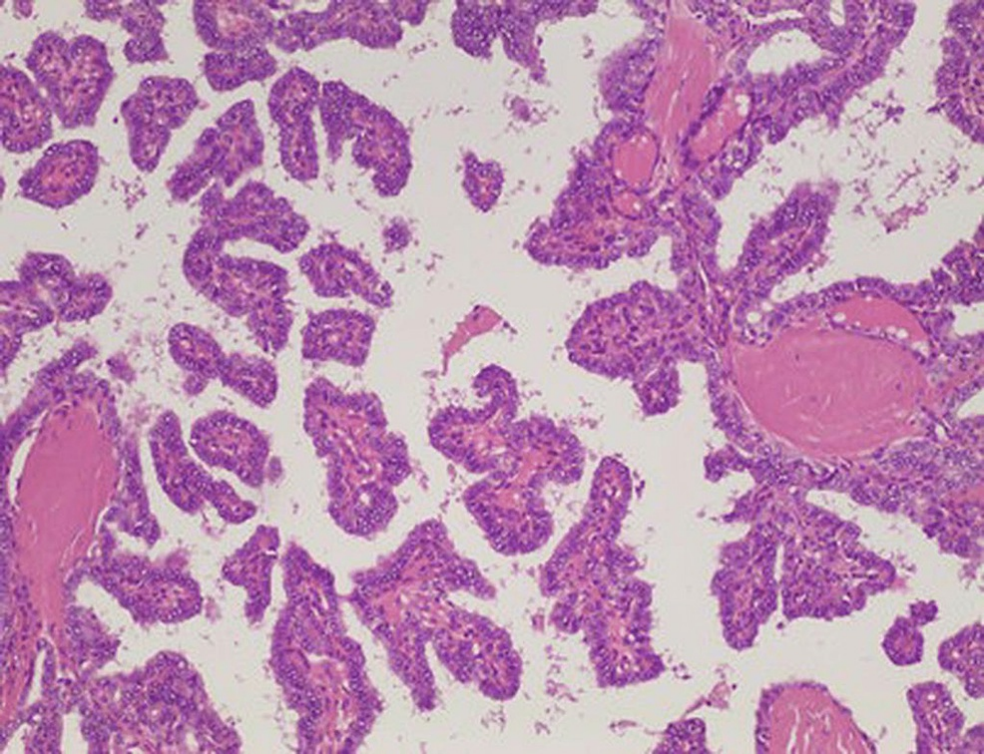
Papillary carcinoma, classic type with typical papillary pattern of growth and nuclear features of papillary thyroid carcinoma. No colloid of Psammoma bodies was present. 100x magnification.

On anatomopathological evaluation, the presence of papillary carcinoma was observed in a cystic lesion of the thyroglossal canal (Figures 3, 4).

**Figure 5:**
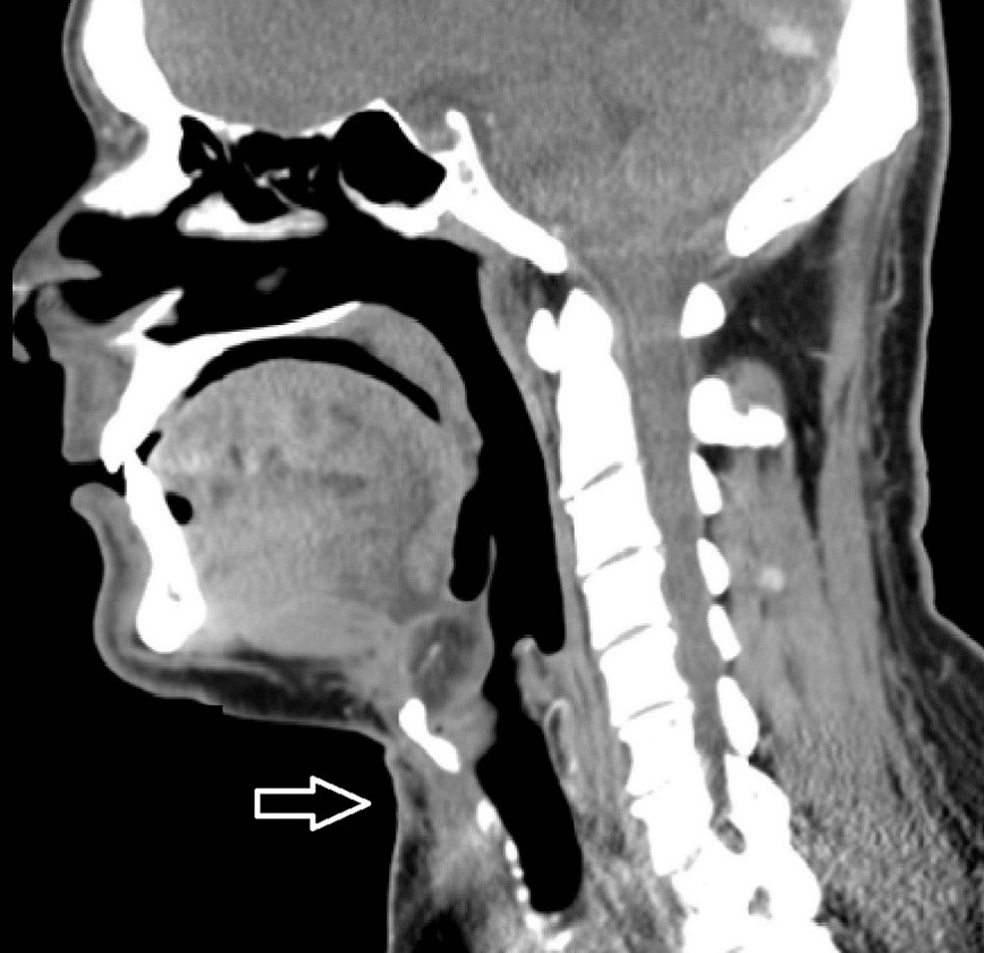
Sagittal CT image of the same patient, one year after surgical treatment, showing removal of the cystic lesion and of the body of the hyoid bone.

The postoperative course was uneventful, with the resolution of symptoms and no complications. Figure 5 shows a CT image of the same patient, one year after surgical treatment, showing the removal of the cystic lesion and of the body of the hyoid bone. At the one-year follow-up, the patient had developed no further symptoms and no signs of local or regional recurrence. The patient remains under surveillance, with ENT consultation every six months and yearly ultrasound and analytical thyroid hormones (thyroid-stimulating hormone (TSH), T4, T3) surveillance.

## Discussion

In approximately 1% of congenital remnants of thyroglossal ducts, malignant transformation is detected [3]. There may be a variety of histologic types of thyroid tumors in cysts, most of which arise from ectopic thyroid tissue [3-5]. Eighty percent of all carcinomas in this context are papillary, followed by "mixed" papillary-follicular carcinomas (8%), and squamous cell carcinomas (6%). Follicular carcinoma presents more often in females, and a small female predominance has been documented in patients with follicular carcinoma within TDC [6-8]. In most cases, patients present with no symptoms, except for neck swelling. The development of carcinoma within TDC does not appear to change its presentation [5]. Page et al. stated that patients with onset of pain, changes in voice, the rapid development of the tumor, weight loss, lymphadenopathy, and respiratory symptoms (such as dyspnea by compression of the airway) suggest malignant transformation, but these are unusual findings [4]. Clinical examination, ultrasound, FNA, and CT findings can help to make a preoperative diagnosis, but most carcinomas are not discovered until surgery and anatomical pathology are performed. FNA cytological examination only diagnoses papillary carcinoma within TDC in two-thirds of the cases [9]. Diagnosis of papillary carcinoma in a TDC raises the question of whether it arises from thyroid tissue present in the wall of the TDC, or if it results from the spread of thyroid carcinoma in the thyroid gland itself. The latter hypothesis raises concern since papillary carcinoma of the thyroid is a multifocal disease. There is concern over this hypothesis because papillary carcinomas of the thyroid are multifocal and have been shown to metastasize without a detectable thyroid lesion. In most benign TDCs, however, ectopic thyroid tissue appears to cause these tumors [10]. In that case, removal of the TDC containing the carcinoma should be enough, with no need to perform a thyroidectomy [3,4].

An evaluation of TDCs for primary carcinoma must differentiate between carcinomas of a clearly discernible TDC or tract, and carcinomas of thyroid glands [5,11]. In most cases, papillary carcinoma within TDC is treated with the Sistrunk procedure, with a reported 95% control rate [7,12,13]. Sistrunk procedure should include en bloc removal of the cyst, part of the hyoid bone, and thyroglossal duct remnants until the tongue base [11]. Thyroidectomy should not be performed unless palpable abnormalities in the gland are observed, or if inadequate follow-up is predicted [3,5]. If routinely performed, thyroidectomy does not seem to have a significant impact on the treatment outcome [4,12,13]. This type of carcinoma usually has an excellent prognosis [7]. Concerning adjuvant treatment, there are no data on its benefit in patients with papillary carcinoma with TDC. However, Plaza et al. proposed in 2005 an algorithm in which suppression hormone therapy was recommended in patients with low-risk disease [14,15]. On the other hand, whenever there is a suspicion of foci in the thyroid gland, large tumor >1.5 cm, nodal disease, age over 45 years, or previous neck irradiation, a total thyroidectomy is followed by I^131 ^ablation and thyroid-stimulating hormone suppression is suggested [16]. In this case, intra-operative examination of the thyroid and postoperative imagiological evaluation showed no abnormalities in the thyroid gland or neck lymph nodes. Thus, no other surgical procedures were proposed, and the patient has not presented recurrence during the follow-up time.

## Conclusions

TDC is the most common congenital anomaly related to the thyroid gland. In a TDC, papillary carcinomas typically present as swellings along the midline of the neck, which move with deglutition and tongue projection. A very low percentage of all TDC cases present with papillary carcinomas. This type of pathology has a good prognosis and can be successfully managed with cyst removal (Sistrunk procedure) and careful follow-up. Thyroidectomy, neck lymph node dissection, or adjuvant treatments are not required in most cases.
